# Pretreatment of UC-MSCs with IFN-α2 improves treatment of liver fibrosis by recruiting neutrophils

**DOI:** 10.1186/s12967-023-04732-0

**Published:** 2023-11-18

**Authors:** Ye Xie, Jia Yao, Mengchao Yan, Yan Lin, Jiayun Wei, Haiping Wang, Yongcui Mao, Pinyan Liu, Xun Li

**Affiliations:** 1 The First Clinical Medical College, Lanzhou University, Lanzhou, China; 2 Department of General Surgery, The First Hospital of Lanzhou University, Lanzhou, China; 3 Key Laboratory of Biotherapy and Regenerative Medicine of Gansu Province, Lanzhou, China

**Keywords:** Cirrhosis, Umbilical cord mesenchymal stem cells, Neutrophils, Microenvironment-induced phenotype

## Abstract

**Background:**

The use of umbilical cord mesenchymal stem cells (UC-MSCs) is a burgeoning method for the treatment of liver cirrhosis. However, the secretory phenotype and regulatory ability of UC-MSCs are easily affected by their microenvironment. Ensuring a specific microenvironment to enhance the UC-MSCs phenotype is a potential strategy for improving their therapeutic efficacy. The aim of this study was to explore therapeutic UC-MSCs phenotypes for improving liver fibrosis.

**Methods:**

RNA-sequencing was used to analyze the response pattern of UC-MSCs after exposure to the serum of cirrhotic patients with HBV. Using immunohistochemistry, quantitative polymerase chain reaction, and immunofluorescence techniques, we evaluated the therapeutic effect of UC-MSCs pretreated with interferon alpha 2 (IFN-α2) (pre-MSCs) in an animal model of cirrhosis. Immunoblotting, ELISA, and other techniques were used to analyze the signaling pathways underlying the IFN-induced changes in UC-MSCs.

**Results:**

UC-MSCs exposed to the serum of patients with hepatitis B-induced cirrhosis showed an enhanced response to type I IFN. The activated type I IFN signal induced the highest secretion of colony-stimulating factor 3 (CSF-3), interleukin (IL)-8, and chemokine (C–C motif) ligand 20 (CCL20) by the UC-MSCs. Pre-MSCs showed a higher therapeutic efficacy than untreated UC-MSCs in an animal model of liver fibrosis. Immunohistochemical analysis revealed that pre-MSCs could recruit neutrophils resulting in an increase in the secretion of matrix metalloprotease 8 that alleviated fibrosis. When neutrophils in animals were depleted, the therapeutic effect of pre-MSCs on fibrosis was inhibited. IFN-α2 altered the secretory phenotype of UC-MSCs by activating phosphorylated signal transducer and activator of transcription 1 and 2 (p-STAT1 and p-STAT2).

**Conclusions:**

Pre-MSCs exhibited enhanced secretion of CSF-3, IL-8, and CCL20 and recruited neutrophils to alleviate fibrosis. This new strategy can improve cell therapy for liver cirrhosis.

**Graphical abstract:**

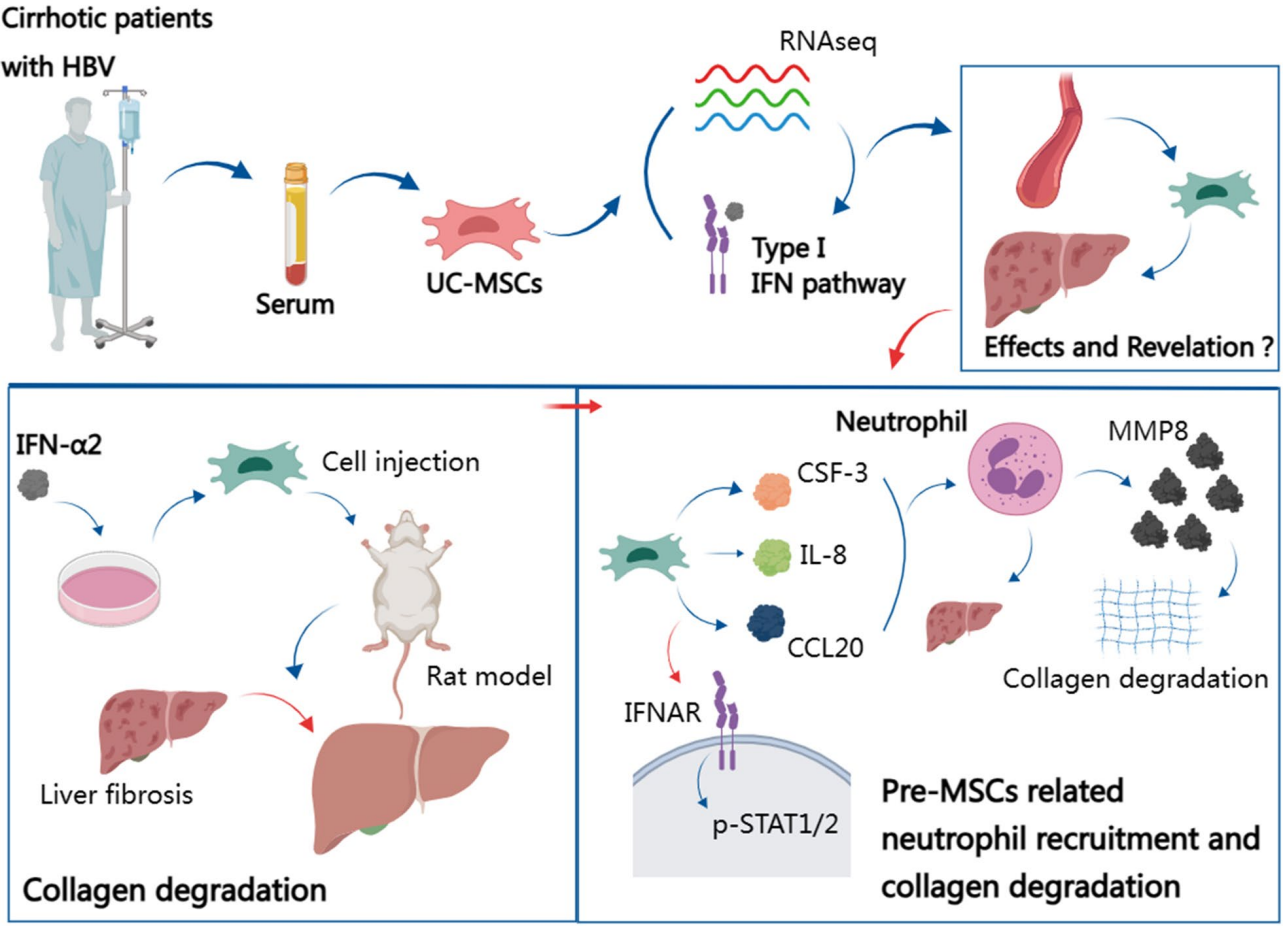

**Supplementary Information:**

The online version contains supplementary material available at 10.1186/s12967-023-04732-0.

## Background

Liver cirrhosis is currently the 11th most frequent cause of death worldwide and accounts for 50% of liver-related deaths. In European and other Western countries, the main causes of liver cirrhosis are alcohol consumption and non-alcoholic fatty liver disease, whereas in China and other Asian countries, the main cause is viral hepatitis B [1]. Chronic and repeated injuries lead to hepatocyte damage, inflammatory cell infiltration, extracellular matrix remodeling, and eventually progress to cirrhosis [2]. Although liver transplantation can be effectively used to treat cirrhosis, the availability of transplants is limited by a shortage of donors. Therefore, a method that can overcome this limitation and effectively alleviate the symptoms of cirrhosis and prolong life is urgently required.

Mesenchymal stem cells (MSCs) are a type of stem cells that can differentiate and regulate immunity and can be isolated and cultured from bone marrow, umbilical cord, fat, and other tissues [3]. Using animal models, MSCs have been shown to effectively reduce fibrosis. In clinical studies, MSCs effectively reduced mortality and improved the quality of life of patients with cirrhosis [4, 5]. Umbilical cord-derived MSCs (UC-MSCs) stand out in the research of exogenous stem cell therapies as they are readily available and have low immunogenicity and strong proliferative ability. Thus, they represent an ideal option in cell therapy for the management of liver cirrhosis [6, 7]. The mechanism underlying the efficacy of UC-MSCs in the treatment of liver cirrhosis involves their paracrine function and immunoregulatory characteristics [8–10]. UC-MSCs can improve the cellular microenvironment in the fibrotic liver through indirect mechanisms, including inhibition of the activation of hepatic stellate cells, regulation of the function of immune cells, generation of a microenvironment that promotes the repair of liver cell damage, and secretion of proangiogenic cytokines and hepatocyte growth factors. These molecules promote angiogenesis and liver regeneration and improve liver function [4, 11].

The secretion and immunoregulatory ability of MSCs are easily affected by culturing conditions. For example, exposure to interferon (IFN)-β can increase the ability of MSCs to inhibit T-cell proliferation [12], and exposure to tumor necrosis factor-α and interleukin (IL)-1β induces them to secrete interleukin (IL)-8 and enhances the recruitment of neutrophils [13]. Additionally, exposure of MSCs to the serum obtained from patients with the disease of interest can improve their immunomodulatory capacity by enhancing their secretion of inflammatory factors to regulate immune cells in that specific microenvironment [14, 15]. Thus, pretreatment of MSCs before an in vivo injection can change their therapeutic phenotype and is a potential strategy to improve the efficacy of cell therapy [16]. However, whether serum from patients with cirrhosis affects the therapeutic phenotype of MSCs and their therapeutic capacity is currently unknown.

In our study, we used the serum of patients with hepatitis B-induced cirrhosis as a pretreatment agent to induce changes in UC-MSCs. After stimulation for 24 h with the serum, the response of UC-MSCs to type I IFNs was enhanced. Furthermore, in an animal model of liver fibrosis, we stimulated UC-MSCs with exogenous IFN-α2 to modulate their immune regulation function and improve their therapeutic effect.

## Methods

### Isolation, purification, and characterization of UC-MSCs

Primary UC-MSCs were extracted from neonatal umbilical cord using the tissue-block attachment method. All the procedures were approved by the Ethics Committee of the First Hospital of Lanzhou University (LDYYLL2020-280). Briefly, Wharton's jelly was separated, and blood was washed with phosphate buffered saline (PBS). The tissue was cut into 1–2 mm^3^ blocks and placed onto the bottom of a T75 culture bottle. An appropriate volume of Dulbecco's modified eagle medium/nutrient mixture F-12 (DMEM/F12) (Biological Industries, Israel) containing 10% fetal bovine serum (FBS; Biological Industries) and 1% streptomycin/penicillin was added. The flask was placed at 37 ℃ in an incubator with 5% CO_2_ for 4 h. After placing the flask upright, culturing was continued. The culture solution was changed every three days, and cell growth was observed. When cell confluency reached 80%, cells were digested with 0.25% trypsin, passaged, and cryopreserved. Sixth-generation cells were selected, and the presence of positive (CD105, CD73, and CD90) and negative (CD45, CD34, CD14, and HLA-DR) markers on their surface was detected using flow cytometry. The isolated primary UC-MSCs met the criteria set by the 2016 Guidelines for Stem Cell Research and Clinical Translation of the International Society for Stem Cell Research [17].

### Flow cytometry

After digestion, the UC-MSCs were washed and resuspended in a single-cell suspension in PBS. Phycoerythrin-conjugated anti-human CD105, CD34, HLA-DR, fluorescein isothiocyanate-conjugated anti-human CD73, CD14, allophycocyanin-conjugated anti-human CD90, and CD45 antibodies (BioLegend, USA) were used for surface marker analysis. The mixture was further incubated in the dark at 4 ℃ for 30 min.

### Serum collection from patients with liver cirrhosis and intervention

Serum from patients with liver cirrhosis (LC serum) from the Department of Infectious Diseases of the First Hospital of Lanzhou University was collected, and the age, sex, and liver biochemical indicators (aspartate aminotransferase [AST], alanine transaminase [ALT], albumin [ALB], and total bilirubin [TBiL]) of the patients were recorded. In addition, the serum of healthy individuals (non-LC serum), matched for age and sex, was collected. Baseline characteristics are presented in Table 1. The collected serum samples from each group (LC and non-LC) were mixed, filtered through a 0.22 µm filter and frozen at − 80 ℃ for subsequent experiments. For the in vitro intervention on UC-MSCs, FBS was replaced with 10% human serum. The intervening medium was removed after 24 h. After rinsing with PBS, the cells were collected, and RNA was extracted for RNA sequencing (RNA-seq). The cells were pretreated with recombinant human IFN-α2 (BioLegend, USA). Different concentrations of IFN-α2 were added (5, 10, 20, 50, or 100 ng/mL), according to the experimental requirements. After 24 h, the IFN-α2-containing medium was removed, DMEM/F12 medium containing 10% FBS was added, and culturing was continued for 24 h. The supernatant, either conditioned medium or IFN-α2-free medium (control), was centrifuged at 1000*g* for 10 min to remove cell fragments and stored at − 80 ℃.

**Table 1:** Baseline characteristics data of patients and healthy donors

Factors	Liver cirrhosis (n = 30)	Healthy donors (n = 10)	*P* value
Age (years)			
Medium	48	46	
Range	26–68	24–72	0.69
Sex (M/F)	18/12	5/5	0.72
AST (U/L)	22.3 ± 5.61	63.5 ± 57.25	0.001
ALT (U/L)	15.9 ± 6.99	47.8 ± 42.80	0.001
ALB (g/L)	43.1 ± 4.21	38.6 ± 7.46	0.03
TBiL (µmol/L)	13.9 ± 5.81	45.8 ± 63.12	0.01
Serum IFN-α2 (pg/mL)	7.4 ± 0.41	55.6 ± 57.43	< 0.001

Data are displayed as mean ± SD

*AST* aspartate aminotransferase, *ALT* alanine transaminase, *ALB* albumin, *TBiL* total bilirubin

### RNA-seq analysis

UC-MSCs were treated with serum for 24 h, and total RNA was extracted. After the purity, concentration, and integrity of the RNA samples were assessed, a cDNA library was established and sequenced using the Illumina platform. Bioinformatics analysis was performed on BMKCloud (www.biocloud.net). Differential expression analysis, functional annotation, and functional enrichment of differentially expressed genes (DEGs) were performed based on the expression of genes in different groups. DEGs were identified using the DESeqR software package (version 1.10.1). The GOstats software package was used to perform Gene Ontology (GO) term enrichment analysis and Kyoto Encyclopedia of Genes and Genomes (KEGG) pathway analysis for the identification of pathways in which DEGs were significantly enriched.

### Design of animal experiments

We purchased four-week-old female Sprague–Dawley rats (120–150 g) from the Lanzhou Veterinary Research Institute of the Chinese Academy of Agricultural Sciences. All animals were housed in a disease-free gas flow cabinet, and free access to food and water was allowed. The animal research program was approved by the Animal Welfare and Ethics Committee of the First Hospital of Lanzhou University (LDYYLL2020-280) and was implemented in accordance with the “Guide for Care and Use of Laboratory Animals”.

A chronic liver fibrosis model was developed by intraperitoneally administering a solution of carbon tetrachloride (CCl_4_) and olive oil at a ratio of 1:1 (v/v), 2 mL/kg, twice a week. The substances were administered for 8 weeks.

#### First experiment

The animals were randomly divided into a Normal group (n = 3), Control group (n = 5), MSC-treated group (n = 5), and Pre-MSC group (n = 5). The normal group was administered an oil injection, and the other three groups were established using the above methods to form a chronic liver fibrosis model. After 8 weeks of establishing the model, the MSC and Pre-MSC groups (UC-MSCs pretreated with INF-α2) received 2 × 10^6^ MSCs or pre-MSCs in 500 µL PBS via their tail vein in the 8th and 9th weeks, respectively. The normal and control groups received 500 µL PBS.

#### Second experiment

Animals with chronic liver fibrosis were randomly divided into two groups: the Anti-PMN group (n = 10), in which rabbit anti-rat polymorphonuclear leukocyte (PMN) anti-serum (Accurate, Westbury, NY) (0.3 mL diluted in 0.2 mL PBS) was injected through the tail vein to deplete neutrophils; and the Isotype group (n = 10) that received an equal volume of normal rabbit serum. The anti-PMN serum was injected every 48 h to maintain a low level of neutrophils in the body until the end of the experiment. All rats received 2 × 10^6^ pretreated UC-MSCs in 500 µL PBS on the 8th and 9th week (once a week). Peripheral blood was collected for Giemsa staining at 24 and 48 h to observe the number of neutrophils in the peripheral blood.

### Isolation, activity, and migration of neutrophils

Rat neutrophils were extracted using a blood neutrophil separation kit (Solarbio, China), according to the instructions of the manufacturer. The isolated neutrophils were resuspended in Roswell Park Memorial Institute 1640 medium containing 10% FBS, seeded at a concentration of 2 × 10^5^/mL in a six-well plate with control or conditioned medium (50%/50%, v/v). Neutrophils were cultured at 37 ℃ and 5% CO_2_ for 3 h and stained with trypan blue to observe their activity. To observe the migration of neutrophils in the Transwell system, neutrophils were suspended in Roswell Park Memorial Institute 1640 medium containing 10% FBS. In total, 250 µL cell suspension containing 1 × 10^6^ cells/mL was added to the upper chamber with 3 μm pores, and conditioned medium was added to the lower chamber. After incubation for the 3 h at 37 ℃ and in an atmosphere containing 5% CO_2_, the number of cells in the lower chamber was counted.

### Hematoxylin and eosin (HE) and sirius red staining

The animal liver tissue was fixed in 10% formalin, routinely dehydrated, embedded in paraffin, and cut into sections (4 μm thick) for HE and Sirius Red staining. The cytoplasm was stained with eosin, collagen fibers were stained with Sirius Red, and nuclei were stained with hematoxylin. Five images per slice were randomly collected to quantify liver fibrosis and analyzed using ImageJ software.

### Immunohistochemistry and immunofluorescence

After the tissue sections were sealed with serum, anti-myeloperoxidase (MPO) antibodies (1:1000; Servicebio, China) were added. The sections were incubated at 4 °C overnight, washed three times with PBS, and treated with horseradish peroxidase (HRP)-conjugated Goat Anti-Rabbit IgG (1:200; Servicebio, China) for 1 h at 25 ℃. After 3,3′-diaminobenzidine staining, hematoxylin was used to stain the nuclei. Immunofluorescence was induced by incubating the sections overnight with anti-matrix metalloproteinase 8 (MMP8) antibody (Proteintech, China) at 4 °C. The sections were subsequently washed with PBS and incubated for 1 h at 25 ℃ with Alexa Fluor-488 and Cy3 combined secondary antibody. We used 4′,6-diamidino-2-phenylindole to stain the nucleus, and specimens were examined using a tissue section digital scanner (3D HISTECH, Hungary).

### Knockdown of IFN α/β receptor subunit 1 (IFNAR1) in UC-MSCs

To obtain UC-MSCs with low IFNAR1 expression, we seeded cells at a density of 1 × 10^5^ cells/mL in six-well plates. After 24 h, we added lentivirus (Genechem, China)-transfected UC-MSCs according to the manufacturer’s instructions. The culture medium was changed after 24 h. When the cells reached 80–90% confluence, we used 1 µg/mL purinomycin to screen and purify the transfected cells and then cultured them for subsequent mRNA and protein analyses. The utilized IFNAR1-short hairpin (sh)RNA sequence was: 5′-CCGGGTTGACTCATTTACACCATTTCTCGAGAAATGGTGTAAATGAGTCAACTTTTTG-3′.

### Gene expression analysis

Total RNA was extracted from cells or tissues using TRIzol (Takara, Japan); the amount of RNA was quantified using Nanodrop (Thermo Fisher Scientific, USA), and cDNA was synthesized using a cDNA synthesis kit (Thermo Fisher Scientific, USA). Specific gene expression was analyzed using a real-time polymerase chain reaction (PCR) detection system (Bio-Rad, USA) according to the instructions of the QuantiNova SYBR Green PCR kit (Qiagen, Hilden, Germany). All gene expression levels were normalized to those of GAPDH or β-actin. The primers used are listed in Additional file 1: Table S1.

### ELISA

The levels of colony-stimulating factor 3 (CSF-3), IL-8, and chemokine (C–C motif) ligand 20 (CCL20) in the cell supernatants were evaluated using human CSF-3 (Dakewe, China), human IL-8 (Dakewe, China), and human CCL20 (Proteintech, USA) ELISA kits, respectively. The level of expression of IFN-α2 in human serum was assessed using a human IFN-α2 precoated ELISA kit (Dakewe). The levels of MMP8 and tissue inhibitors of metalloproteinase 1 (TIMP1) in the liver tissue homogenate of rats were measured using a rat MMP8 ELISA kit (JL21063; China) and rat TIMP1 ELISA kit (JL12717; China), respectively. All assays were performed in accordance with the manufacturer’s protocol.

### Western blotting

Cells were lysed with RIPA lysis buffer and incubated for 2 min with an ultrasonic crusher (Scientz, China). A precooled overspeed centrifuge (Thermo Fisher Scientific, USA) was used to centrifuge the mix at 4 ℃ and 12,000*g* for 15 min. The supernatant was extracted and a bicinchoninic acid protein quantitative kit (Beyotime, China) was used to quantify the protein concentration. The proteins were separated on a 10% gel using sodium dodecyl-sulfate polyacrylamide gel electrophoresis and electrotransferred to polyvinylidene fluoride membranes (Merck, USA). The membranes were sealed with a protein-free fast-sealing solution (Servicebio, China) at 25 ℃ for 10 min. After incubation with the corresponding primary antibody at 4 ℃ overnight and with HRP-conjugated secondary antibodies (1:5000; SAB, USA) at 25 °C for 1 h, the proteins were visualized using western blotting. The primary antibodies were anti-IFNAR1 (1:500; Proteintech, USA), β-actin (1:1000; Servicebio, China), anti-STAT1 (1:2000; Proteintech, USA), phospho-STAT1 (Tyr701; 1:1000; Proteintech, USA), anti-STAT2 (1:500; Proteintech, USA), phospho-STAT2 (Tyr690; 1:1000; SAB, USA), anti-STAT3 (1:500; SAB, USA), and phospho-STAT3 (Try705; (1:500; SAB, USA).

### Degradation rate of type I collagen (COL I)

Extract 150ug of total protein from liver tissue and mix with incubation solution 1:4 (v/v). The protein extraction and quantification methods are consistent with western blotting. A mixture of protein and incubation solution was incubated at 37 ℃ for 0 and 48 h. Collect the supernatant and use western blotting to determine the content of COL I (Anti-Collagen I; 1:1000; PTMBIO, China). The activity of MMP8 was indicated by evaluating the degradation rate of COL I after incubation.

### UC-MSCs migration in Transwell system

Firstly, UC-MSCs were pretreated with 5 ng/mL IFN-α2 for 24 h. UC-MSCs cultured without IFN-α2 were used as the control. After digestion, cells were resuspended in DMEM/F12 medium containing 5% FBS, and the cell density was adjusted to 2 × 10^5^ cells/mL. Then, 100 μL of the solution was placed in the upper chamber of the Transwell system with 8.0 μm pores (Corning, USA). Add DMEM/F12 serum (600 μL) containing 30% FBS was add to the lower chamber. After 24 h, the upper chamber was removed and the sample was stained with 1% crystal violet to count the number of positive cells.

### Signal transducer and activator of transcription 1 (STAT1) inhibition

Fludarabine (20 µM; MedChemExpress, USA) was added to the UC-MSC medium to inhibit the activation of STAT1 and STAT1-dependent gene transcription. The cell supernatant was collected to determine CSF-3, IL-8, and CCL20 levels and standardize with the corresponding cell number.

### Statistical analysis

Multi-group differences were compared using ANOVA, and differences between two groups were compared using Student’s t-test. All data were tested for normality and homogeneity of variance. All *P* values < 0.05 were considered statistically significant. GraphPad Prism 8.0 and SPSS 20.0 were used to process the data and draw graphs.

## Results

### Isolation and characterization of UC-MSCs

Primary UC-MSCs were obtained from the adherent tissue mass in approximately 10 days. They grew in a dense spindle-shaped and side-by-side manner, with a clear single-layer nucleus (Additional file 1: Fig. S1A). After continuous passage, cells from the sixth-generation were selected for flow cytometry. The results indicated that in these cells, the levels of expression of the positive markers CD105, CD73, and CD90 were over 95%, and those of the negative markers CD34, CD45, CD14, and HLA-DR were less than 2% (Additional file 1: Fig. S1B). Therefore, the isolated primary UC-MSCs met the criteria set by the International Society for Cellular Therapy [17].

### LC serum enhances the response of UC-MSCs to type I IFN

**Fig. 1:**
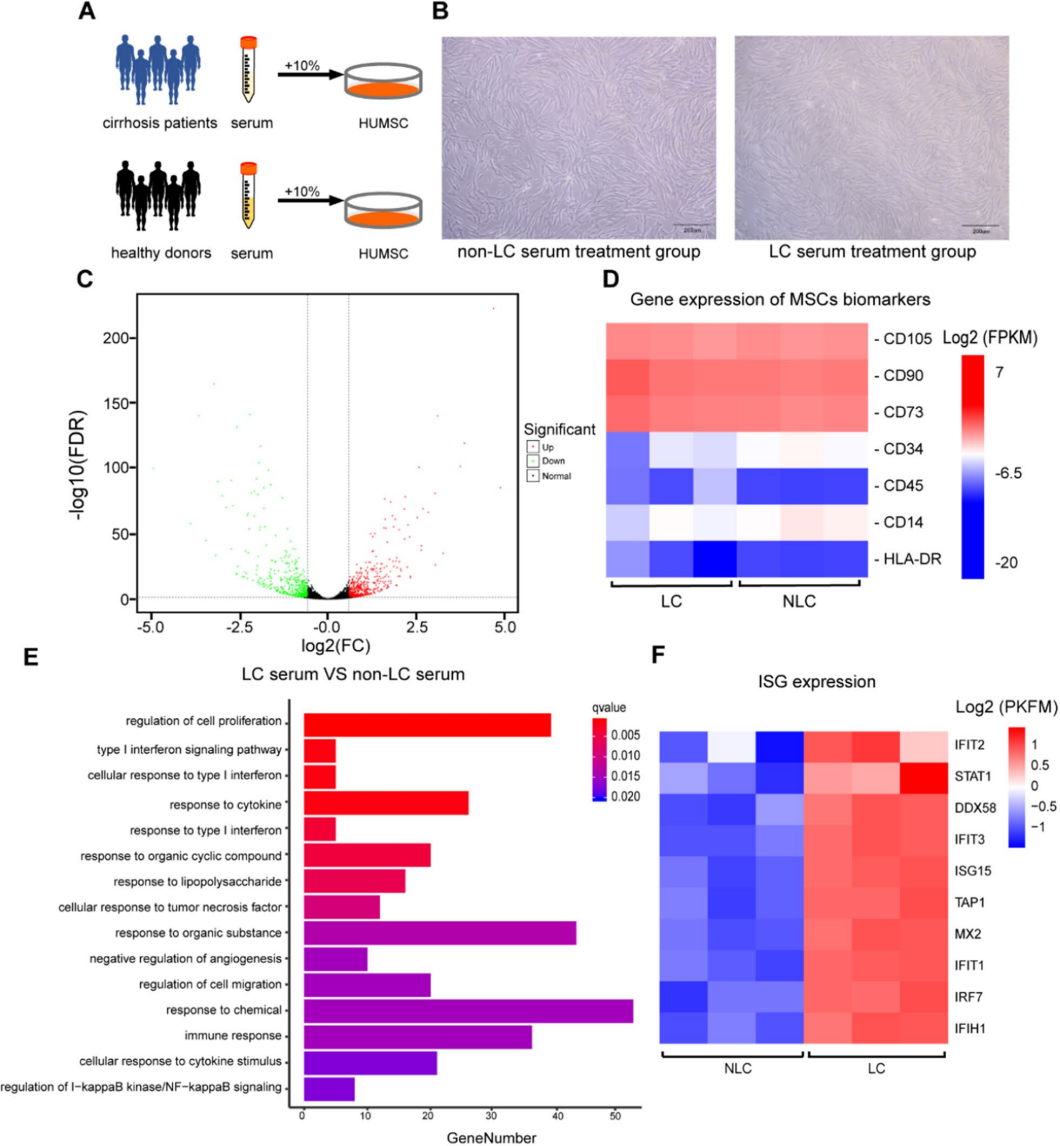
Analysis of phenotype of UC-MSCs in different serum.** A** Schematic diagram of different types of serum intervention UC-MSCs. **B** Cell morphology after intervention with non-LC serum and LC serum. Scale bars, 200 μm **C** Volcano plot showing the up- (red) or downregulated (green) genes between the non-LC serum treatment group (non-LC group) and the LC serum treatment group (LC group). **D** Heat map of gene expression of MSCs biomakers between LC group and non-LC group. **E** GO annotated statistics based on biological processes of differentially expressed genes between LC group and non-LC group. **F** Heat map of gene expression of ISG between LC group and non-LC group

UC-MSCs were treated with LC or non-LC serum for 24 h. No obvious changes in the cell phenotype were observed under a microscope (Fig. 1A, B). We then analyzed and compared the transcriptome of the two groups. Using a fold change ≥ 1.5 and a false discovery rate < 0.05 as the screening criteria, we screened 987 DEGs between the two groups, including 534 and 453 genes with upregulated and downregulated expression, respectively (Fig. 1C). However, there was no difference in the gene expression of the UC-MSC biomarkers (Fig. 1D). To better understand the specific functional enrichment pathways of the DEGs, biological function enrichment analysis was performed. The results showed that LC serum enhanced the response of UC-MSCs to type I IFN and activated the type I IFN response pathway (Fig. 1E). Furthermore, the expression of IFN-stimulated gene (ISG) activated by IFN also increased (Fig. 1F).

### Changes in the expression of cytokines in UC-MSCs treated with type I IFN-α2

**Fig. 2:**
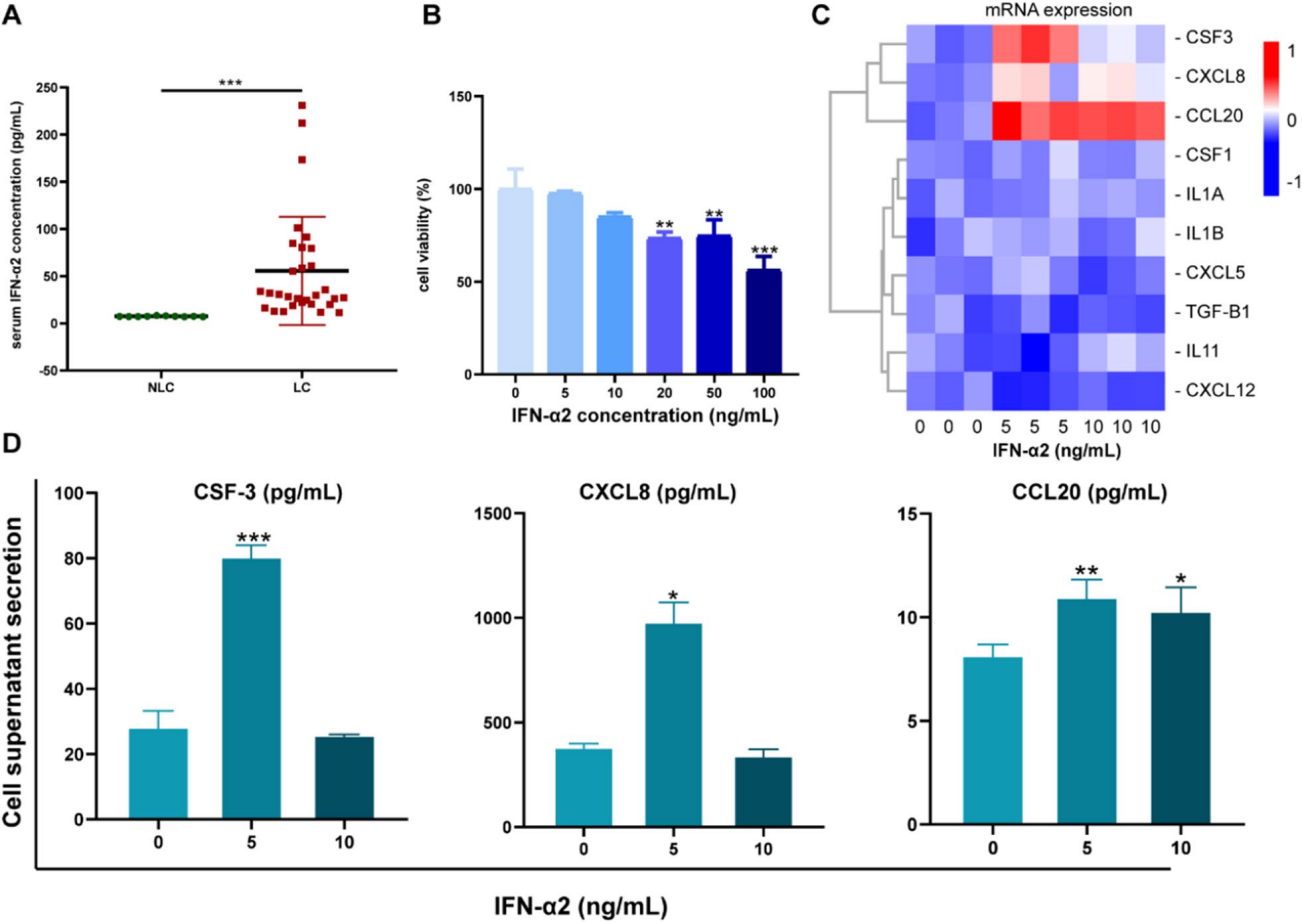
IFN-α2 induced changes inflammatory factor expression of UC-MSCs.** A** Analysis of IFN-α2 content in serum from patients with liver cirrhosis (n = 10) and healthy donors (n = 30). **B** Effects of different concentrations on the activity of UC-MSCs. **C** Relative mRNA expression levels of inflammatory factors in UC-MSCs treated with 5 ng/mL IFN-α2 and 10 ng/mL IFN-α2. **D** ELISA Analysis of CCL2 protein secretion levels in cell suprnatant after treated with 5 ng/mL IFN-α2 and 10 ng/mL IFN-α2. *P < 0.05, **P < 0.01, ***P < 0.001

The type I IFN family is composed of 16 members, namely 12 IFN-α subtypes, IFN-β, IFN-ε, IFN-κ, and IFN-ω [18]. After infection with hepatitis B virus, the expression of all subtypes of type I IFNs increased, including that of IFN-α2, which has a strong antiviral effect and has been used in the antiviral treatment of hepatitis B [19, 20]. Thus, we selected IFN-α2 as an activator of IFN. We collected serum from patients with hepatitis B and healthy donors and measured the serum levels of IFN-α2. The results revealed that, compared to healthy donors, patients with hepatitis B expressed higher levels of IFN-α2 (Fig. 2A). Additionally, the RNA-seq results revealed changes in the secretion of inflammatory factors by the UC-MSCs exposed to serum (Additional file 1: Table S2). Next, we used exogenous IFN-α2 as a pretreatment agent to explore the changes observed in UC-MSCs. First, UC-MSCs were stimulated with different concentrations of IFN-α2 for 24 h, and their cellular activity was observed. The results indicated that cell proliferation was inhibited when IFN-α2 concentration was 20 ng/mL (Fig. 2B). Thus, in the follow-up experiments, we used IFN-α2 at concentrations of 5 and 10 ng/mL. IFN-α2 induced the secretion of inflammatory cytokines by UC-MSCs, especially when the concentration of IFN-α2 was 5 ng/mL. Thus, the levels of expression of CSF-3, IL-8, and CCL20 were significantly higher in UC-MSCs treated with IFN-α2 than in controls (*P* < 0.001, *P* = 0.012, and *P* = 0.04) (Fig. 2C). The secretion of cytokines in the cell supernatant showed the same trend (Fig. 2D). The results showed that 5 ng/mL IFN-α2 had the best inducing effect on UC-MSCs to produce CSF-3, IL-8, and CCL20. These results suggest that our pre-MSCs may play a role in immune regulation.

In addition, we used flow cytometry to support the pre-MSCs biomarkers without any changes. The result indicated that UC-MSCs met the criteria set by the International Society for Cellular Therapy [17] (Additional file 1: Fig. S2).

### Pretreatment of UC-MSCs with IFN-α2 can improve their therapeutic effect

We observed the therapeutic effect of UC-MSCs pretreated with 5 ng/mL IFN-α2 in rats with liver fibrosis. In previous studies on MSC therapy for liver diseases, the recruitment of MSCs in the liver is the basis for the therapeutic effect, which is supported by increasing evidence. For example, in animal experiments, Shannon et al. found that in steady-state bodies, MSC mainly concentrated in the liver on day 7 and disappeared after 14 days [21]. At the same time, in injured bodies, MSCs will spontaneously migrate to the site of inflammation and injury [22]. Specifically, in an animal model of liver fibrosis, staining and localization of labeled MSCs in liver tissue demonstrated that they are indeed recruited into the liver. [23–25]. Therefore, our research is based on previous evidence. Before observing the therapeutic effect of fibrosis in vivo, we evaluated the migration changes in UC-MSCs pretreated with IFN-α2 in vitro. The results showed that there was no significant difference in the positive cells between pre-MSCs and MSCs, indicating that IFN-α2 did not affect the migration of UC-MSCs (Additional file 1: Fig. S3).

**Fig. 3:**
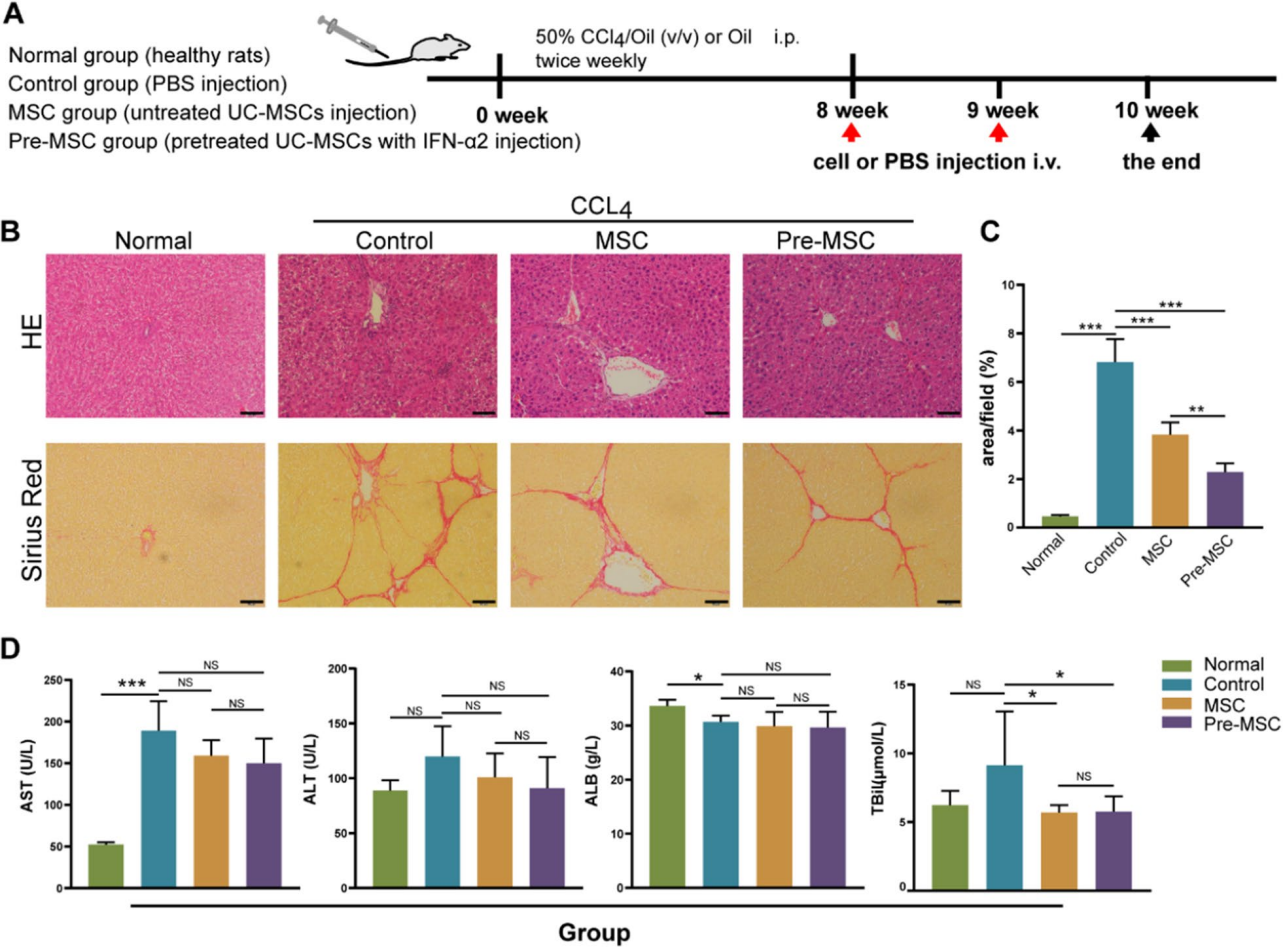
Evaluation of the effect of MSC with different treatment on rat liver fibrosis.** A** Animal modeling and treating diagram. **B** HE staining and Sirius Red staining of liver tissues in Normal (n = 3), Control (n = 5), MSC (n = 5), and Pre-MSC groups (n = 5). Scale bars, 50 μm **C** The positive area of Sirius Red staining statistics in Normal, Control, MSC, and Pre-MSC groups. **D** Biochemical index (AST, ALT, ALB, TBil) checked in Normal, Control, MSC, and Pre-MSC groups. *P < 0.05, **P < 0.01, ***P < 0.001

After the fibrosis model was successfully established, the tested cells were injected once a week for two weeks. One week after the last treatment, we assessed the pathological changes in the liver and biochemical indicators (Fig. 3A). HE staining revealed that the livers of animals in the Control group had obvious structural damage and presented pseudo-lobule formation. The hepatocytes exhibited different degrees of degeneration and necrosis (Fig. 3B). After two weeks of repeated cell therapy, Sirius Red staining of the liver revealed that, compared to the Control group, cell-treated groups presented a substantially lower deposition of collagen fibers, and the Pre-MSC group had a better effect than the MSC group (Fig. 3C). The indicators in the serum showed that, compared with that in the Normal group, AST in the Control group was significantly increased, while ALB was significantly reduced. Although the average levels of ALT and TBiL in the Control group were higher than those in the Normal group, there was no significant difference. After treatment, both the MSC and Pre-MSC groups showed a significant decrease in TBiL compared to that in the Control group. The average level of AST and ALT decreased, but there was no statistical difference (*P* > 0.5) (Fig. 3D).

**Fig. 4:**
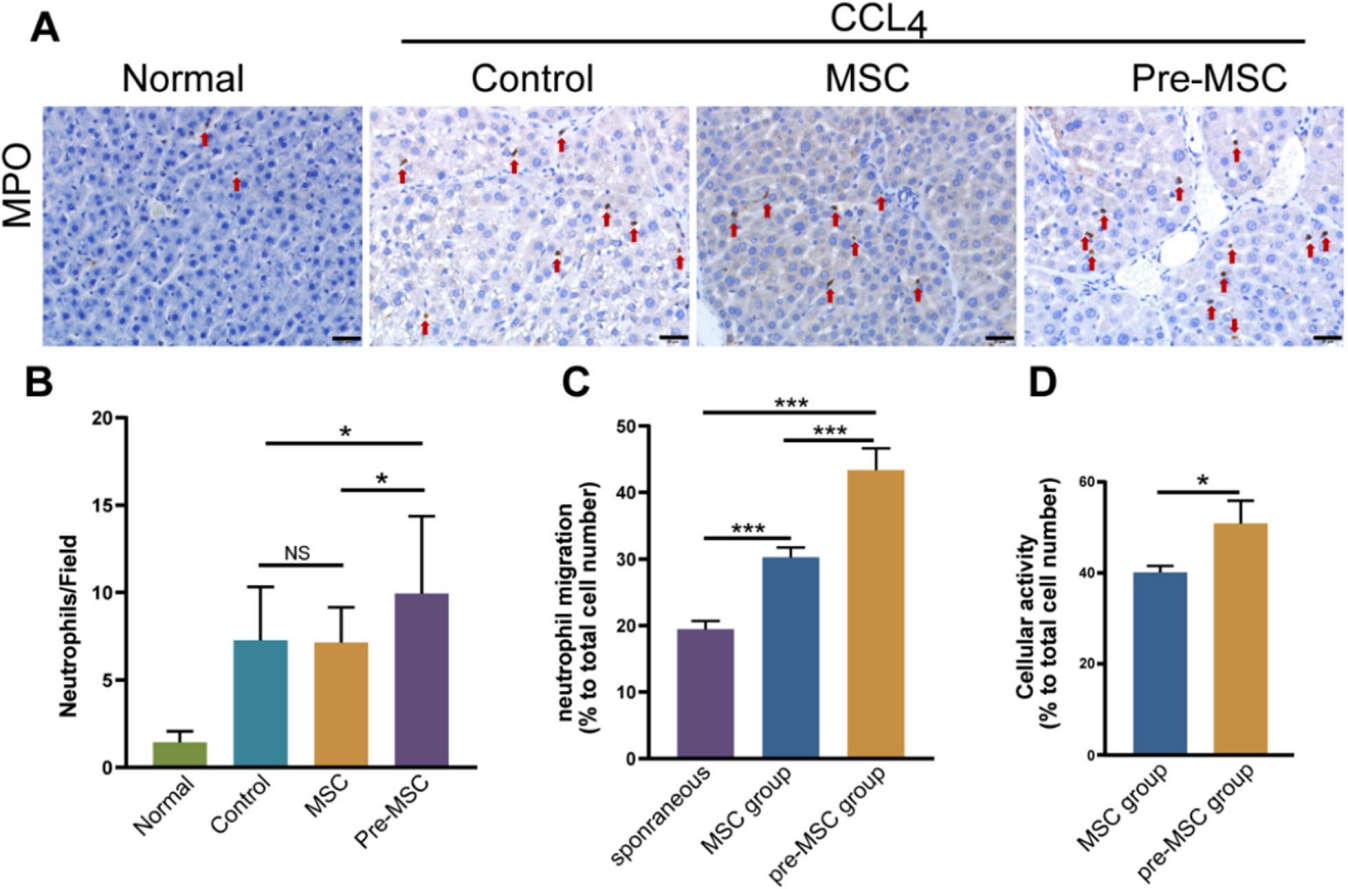
Effect of UC-MSCs pretreated with IFN-α2 on neutrophils. **A** MPO positive neutrophils of liver tissues by IHC in each group. Red arrows indicate stained neutrophils. Scale bars, 20 μm. **B** MPO positive neutrophils count of each field. Scale bars, 20 μm. **C** Neutrophil migration in UC-MSCs condition medium with different treatments. **D** Neutrophils activity in UC-MSCs condition medium with different treatments. *P < 0.05, **P < 0.01, ***P < 0.001

Combining the secretion characteristics (high expression of CSF-3, IL-8, and CCL20; Fig. 2D) of UC-MSC using IFN-α2 pretreatment, we consider that neutrophils are a common target for these different cytokines. Therefore, we further evaluated the infiltration of neutrophils in the liver. The Pre-MSC group presented an increase in neutrophils compared with the control and MSC groups, while the number of neutrophils in the MSC group did not change compared to that in the Control group (Fig. 4A, B). Next, we examined the in vitro migration and activity of neutrophils exposed to the supernatant of pre-MSCs using a Transwell assay. Compared with the spontaneous migration group, the neutrophils exposed to the supernatant of UC-MSCs presented enhanced migration. Furthermore, the supernatant of the pretreated UC-MSCs had the strongest induction effect on the neutrophils (Fig. 4C). The assessment of cell activity indicated that the activity of neutrophils exposed to the pretreated UC-MSC medium was significantly higher than that of neutrophils exposed to the untreated UC-MSC medium (*P* < 0.05) (Fig. 4D). In vitro experiments demonstrated that pretreated UC-MSCs may promote the migration of liver neutrophils and enhance their activity.

### Enhanced therapeutic efficacy of UC-MSCs pretreated with IFN-α2 in liver fibrosis is based on increased recruitment of neutrophils

**Fig. 5:**
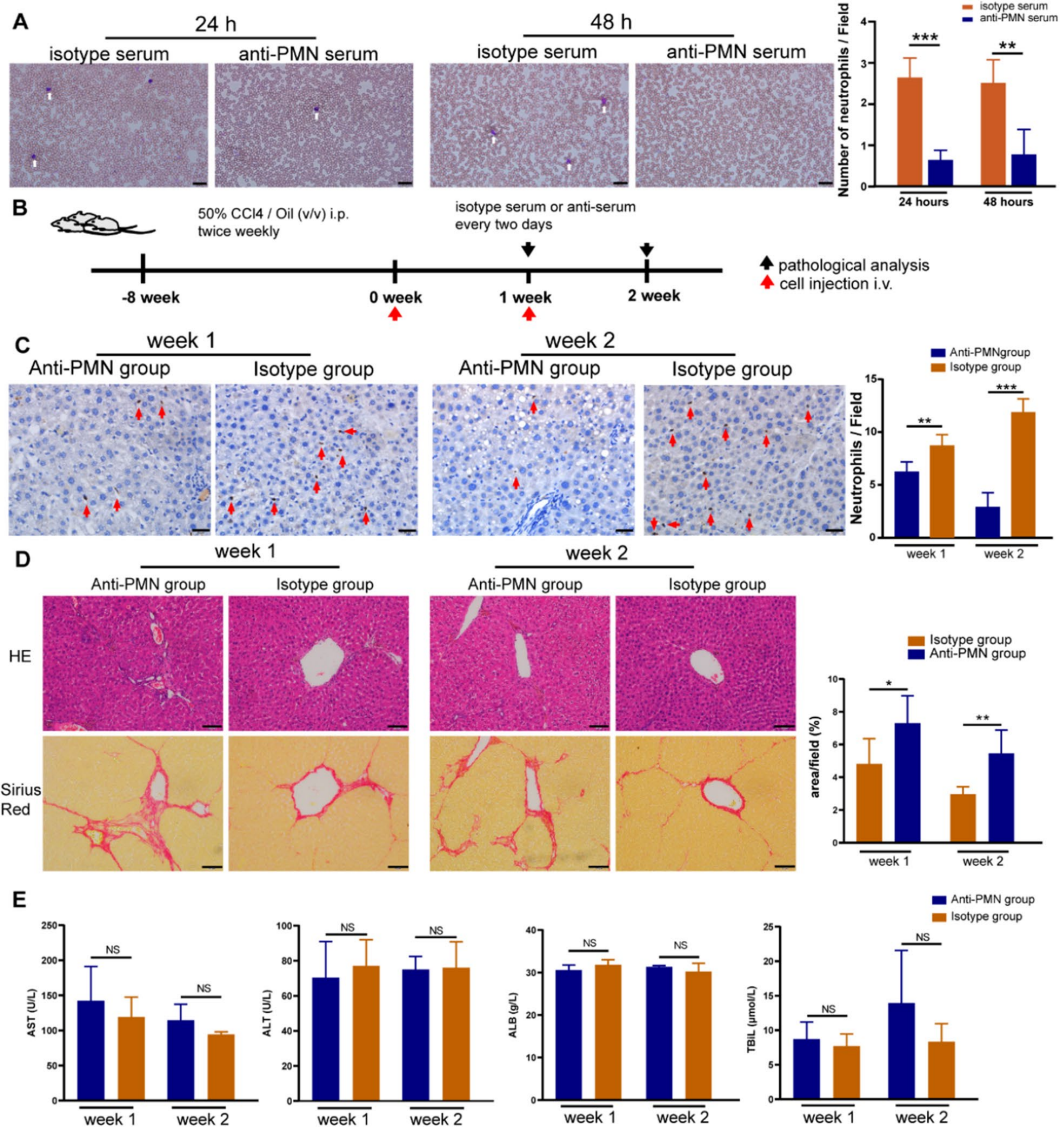
Pretreated UC-MSCs with IFN-α2 enhanced neutrophils infiltration into liver. **A** Representative Giemsa staining of blood smears of rat in Anti-PMN group and Isotype group (Left) and number of neutrophils per field counted (Right). White arrows indicate stained neutrophils. Scale bars, 20 μm. n = 5. **B** Animal modeling and treating diagram. **C** MPO positive neutrophils of liver tissues by IHC in each group (Left) and MPO positive neutrophils count of each field (Right). Red arrows indicate stained neutrophils. Scale bars, 20 μm. **D** HE staining and Sirius Red staining of liver tissues (Left) and the positive area of Sirius Red staining statistics (Right). Scale bars, 50 μm. **E** Biochemical index (AST, ALT, ALB, TBil) in different groups. Number of each group ≥ 3. *P < 0.05, **P < 0.01, ***P < 0.001

To further verify whether the improvement of the therapeutic effect of pre-MSCs was mediated by the recruitment of neutrophils, we used anti-PMN serum to deplete the animals of neutrophils. The anti-PMN serum effectively neutralized neutrophils, and the effect was maintained for at least 48 h (Fig. 5A). Therefore, anti-PMN serum was injected every 48 h until the end of the experiment (Fig. 5A). At the end of the modeling period (8 weeks), we administered pre-MSCs through the tail vein twice a week. The animals were sacrificed after the first or second week of treatment (Fig. 5B). The number of neutrophils in the liver of animals from the Anti-PMN group was significantly lower than that in the liver of animals from the Isotype group (Fig. 5C). Furthermore, HE staining indicated that the degree of hepatocyte necrosis in the Isotype group was lower than that in the Anti-PMN group; Sirius Red staining revealed that the degree of fibrosis in the Isotype group was lower than that in the Anti-PMN group in both the first and second weeks (Fig. 5D). However, there was no significant difference in the levels of ALT, AST, ALB, and TBiL between the two groups (Fig. 5E).

**Fig. 6:**
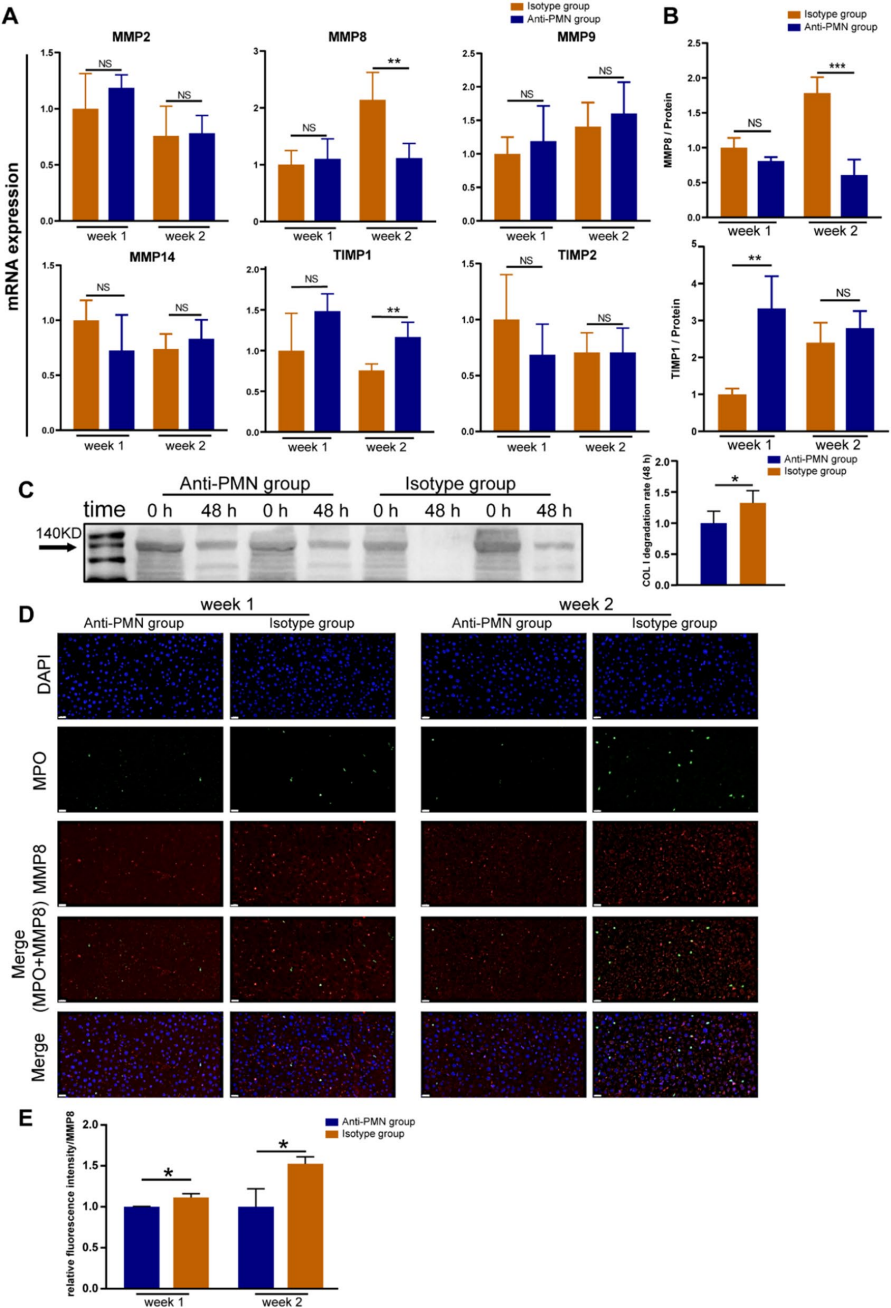
expression patterns of MMPs and TIMPs in liver. **A** mRNA expression of MMP2, MMP8, MMP9, MMP14, TIMP1, and TIMP2 in liver. **B** Relative protein expression of MMP8 and TIMP1. **C** Degradation rate of COL 1; Western blot (Right) and relative degradation rate (Left) for the COL 1 in tissues. **D** Immunofluorescence co-localization of MMP8 (red) and neutrophils (green) in liver. Scale bars, 20 μm. **E** The bar graph shows the mean fluorescence intensity of MMP8. *P < 0.05, **P < 0.01, ***P < 0.001

Neutrophils can secrete enzymes related to collagen degradation, which may be the reason why the increase in neutrophils in the liver can alleviate fibrosis [26, 27]. Therefore, we measured the expression of matrix metalloproteinases (MMP2, MMP3, MMP8, MMP9, MMP13, and MMP14) and inhibitors (TIMP1 and TIMP2) in liver tissues through ELISA. The expression of MMP3 and MMP13 was too low, so the data are not displayed here. After the second treatment, the expression of MMP8 in the isotype serum group was significantly higher than that in the anti-serum group. Interestingly, the expression of TIMP1 in the Anti-PMN group was higher than that in the Isotype group (Fig. 6A). We measured MMP8 and TIMP1 levels in the tissues using ELISA. The secretion of MMP8 showed the same trend as the expression of genes, whereas the secretion of TIMP1 was different after the first, but not after the second, treatment (Fig. 6B). MMP8 is a major member of the MMP family collagenase group and a key enzyme in the degradation of COL I. Therefore, measuring the degradation rate of COL I can reflect the activity of MMP8. We compared the degradation rate of COL I by MMP8 enzymes in the liver after the second treatment in the Anti-PNM group and Isotype group. The results showed that COL I was significantly reduced after incubation for 48 h, and the degradation rate of the Isotype group was significantly higher than that of the Anti-PMN group (Fig. 6C). Using tissue immunofluorescence, we studied the distribution of MMP8 in the liver in different groups. Anti-MPO antibodies were used to locate neutrophils (green), and anti-MMP8 antibodies were used to mark MMP8 proteins (red). As shown in Fig. 6D, E, after the injection of pre-MSCs, the distribution of neutrophils in the liver increased in the animals in the Isotype group, and the surrounding MMP8 was diffusely distributed; Conversely, a small number of neutrophils and weak immunofluorescence were observed in the liver of the Anti-PMN group.

### IFN-α2 regulated UC-MSCs secrete neutrophil-related factors IL-8, CSF-3, and CCL20 by activating STAT1 and STAT2 phosphorylation

**Fig. 7:**
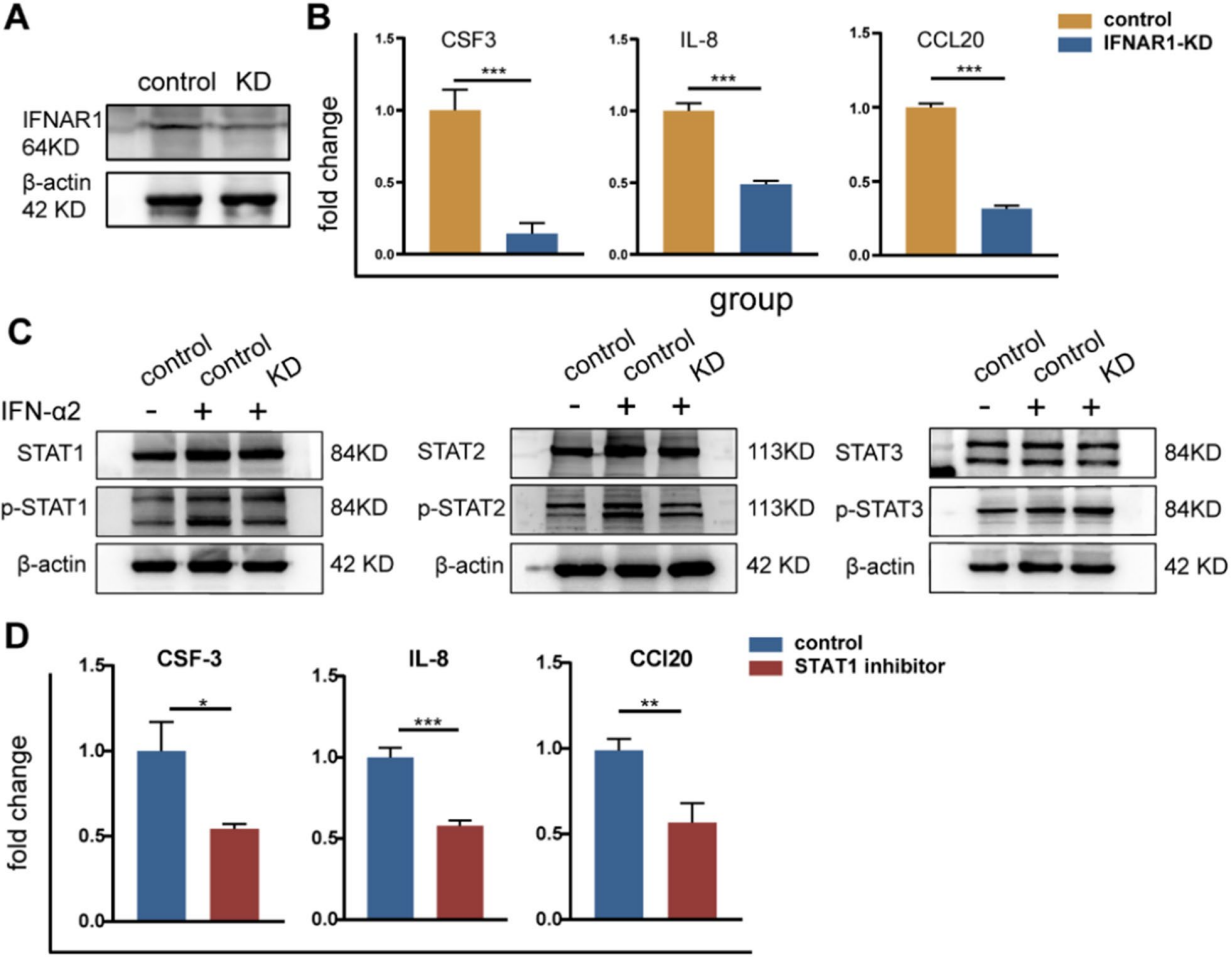
IFN-α2 regulates the secretion of IL-8, CSF-3 and CCL20 by activating the p-STAT1 and p-STAT2 through IFNAR1 receptor. **A** The protein expression of IFNAR1 in control group and IFNAR1-KD group. **B** Fold change of CSF-3, IL-8, and CCL20 in the cell supernatant by ELISA. **C** Protein expression with or without adding IFN-α2 in control UC-MSCs and IFNAR1-KD UC-MSCs. **D** Fold change of CSF-3, IL-8, and CCL20 in the cell supernatant with or without adding Fludarabine by ELISA. *P < 0.05, **P < 0.01, ***P < 0.001. *KD* knockdown

To explore the mechanism by which IFN-α2 regulates the secretion of IL-8, CSF-3, and CCL20 by UC-MSCs, we used lentivirus transfection to knockdown the IFN-α2 receptor IFNAR1 (Fig. 7A). The secretion of IL-8, CSF-3, and CCL20 in the cell supernatant decreased after IFNAR1 knockdown (Fig. 7B). The JAK-STAT pathway is a classic pathway that is activated in response to IFN induction [28]. Therefore, we observed the protein expression of STAT1/2/3 in the presence of IFN-α2. The results showed that, after IFN-α2 pretreatment, STAT1 and STAT2 expression in UC-MSCs remained unchanged, but the levels of p-STAT1 and p-STAT2 increased compared to those in the control group. The levels of STAT3 and p-STAT3 did not change (Fig. 7C). When IFNAR1 was knocked down, the effect of IFN-α2 on p-STAT1 and p-STAT2 decreased (Fig. 7C). This indicates that IFN-α2 regulates the secretion of IL-8, CSF-3, and CCL20 through the activation of IFNAR1 and, subsequently, of p-STAT1 and p-STAT2. When we used fludarabine, a STAT1 inhibitor, the expression of IL-8, CSF-3, and CCL20 in the cell supernatant decreased, indicating that blocking the STAT1 signaling pathway can weaken the secretion of cytokines by UC-MSCs after stimulated with IFN-α2 (Fig. 7D).

## Discussion

MSCs have shown immense potential in the treatment of liver fibrosis. Changes in secreted active substances in response to pressures at the local microenvironment are the main mechanism by which MSCs affect adjacent cells and tissues—paracrine mechanism [29]. An increasing number of studies have confirmed that a group of MSC-secreted factors plays a role in immune regulation and in anti-inflammatory and anti-apoptotic processes, promoting tissue repair, nutrition, and other functions, and may be the main role bearers in the treatment of liver diseases [30–32]. However, the composition of the secreted group is easily affected by the culture conditions and changes. This characteristic, that is, profile changes in response to local pressure, can be used at the pretreatment stage to obtain better therapeutic phenotypes [33–35]. Our center has been carrying out clinical treatments for liver fibrosis using UC-MSCs for many years. We have noticed that after administration by infusion, the cells first come in contact with the patient's serum. However, the responses of UC-MSCs to the serum of patients with disease were unknown. Therefore, we first used serum from patients with hepatitis B-induced cirrhosis to activate UC-MSCs in vitro and examined how to improve the therapeutic phenotype of UC-MSCs by observing their response to this treatment. We found that the serum of patients with disease can enhance the response of UC-MSCs to type I IFN and activate the type I IFN response pathway.

Besides its antiviral function, type I IFN has shown immunomodulatory effects [36]. These effects occur through activation of its receptor (IFNAR), which is expressed by almost all cells [37, 38]. This further activates the classic JAK-STAT signaling pathway [36] Next, p-STAT1 and p-STAT2 recruit IRF9 to form a stable IFN-stimulated gene factor 3 complex in the nucleus, bind to the target DNA sequence, and regulate gene expression [36]. In our study, in response to type I IFN, we observed activation of p-STAT1 and p-STAT2 and enhanced secretion of IL-8, CSF-3, and CCL20. IL-8 is a powerful chemokine of neutrophils [39, 40]. CSF-3 is a granulocyte colony stimulating factor that induces and regulates the proliferation and differentiation of neutrophils [41]. CCL20, too, has been reported to be involved in the activation of neutrophils [42]. Neutrophils can participate not only in inflammatory reactions, such as infection, but also in tissue repair and fiber degradation [43, 44]. Therefore, we targeted the ability of UC-MSCs to regulate neutrophils and found that the liver fibrosis in the Pre-MSC group was markedly improved and accompanied by the recruitment of more neutrophils in the liver. Therefore, we considered that pretreated UC-MSCs may modify cytokine secretion and recruit neutrophils to reduce collagen.

Neutrophils are inflammatory cells induced by cell apoptosis and necrosis during the formation of liver fibrosis. Together with macrophages, dendritic cells, and lymphocytes, they form a chronic inflammatory microenvironment [45]. Some studies have pointed out that the presence of elastase in neutrophils and that of reactive oxygen species released by the same cells can aggravate liver injury [46, 47]. In addition, in a model of steatohepatitic cirrhosis, neutrophils were shown to communicate with and activate hepatic stellate cells; furthermore, an increase in the survival period of neutrophils was observed. All these elements aggravate fibrosis [48]. However, the role of neutrophils in the process of fibrosis repair cannot be ignored. In cholestatic liver injury, depletion of neutrophils impedes the degradation of early collagen. Neutrophils can secrete enzymes related to collagen degradation, which may explain why the increase in neutrophils in the liver ameliorates fibrosis [49]. Similarly, in the CCL4-induced fibrosis model, an increase in the number of neutrophils inhibits the aggravation of chronic liver injury induced by chemical inducers [50], which supports our findings.

In our study, we also found that neutrophil depletion weakened the therapeutic effect of UC-MSCs, possibly owing to a decrease in the level of MMP8 in the liver. MMP8 is the main member of the collagenase group in the MMP family of neutral proteases and is mainly produced by neutrophils. MMP8 can cleave type I–III collagen, mediating the initial steps of collagen degradation [51, 52]. Collagen is the main structural component of the extracellular matrix. When liver fibrosis occurs, there is an increase in the deposition of extracellular matrix and a lack of degradation. Therefore, our hypothesis was validated based on our determination of MMP8 expression and MMP8 in the liver (Fig. 6B, C). Furthermore, it is worth noting that treatment is improved by collecting neutrophils, which is part of the reason why the pretreated UC-MSCs play a role and relates to our observation that the untreated UC-MSCs have a certain effect on the fibrosis (Fig. 3B).

In this study, we measured the levels of MMPs and TIMPs secreted by neutrophils to reveal the underlying treatment mechanism. Some studies have suggested that the damage-repair ability of neutrophils depends on their synergy with anti-inflammatory macrophages [53, 54]. The various mechanisms by which neutrophils exert their effects in liver injury/fibrosis require further research.

IL-8 belongs to the CXC chemokine superfamily and acts on neutrophils through CXCR1 and CXCR2. IL-8 expression is higher in the serum and tumor microenvironment of most patients with tumors compared with that in healthy individuals. IL-8 in the tumor microenvironment is produced jointly by cancer cells, immune cells infiltrating the tumor, and fibroblasts [55]. IL-8 promotes tumor progression in liver cancer by promoting tumor cell proliferation and epithelial–mesenchymal transition [56]. In addition, IL-8 promotes the production of tumor cell survival niches, including by promoting angiogenesis and the tumor cell glycolysis–related immunosuppressive microenvironment [57, 58]. Conversely, in tumor metastasis models, IL-8 exerts anti-tumor effects by recruiting neutrophils, mainly by producing reactive oxygen that directly kills tumor cells [59], or by activating unconventional T cells to promote anti-tumor immune responses [60]. Therefore, IL-8 plays a dual role in tumors, and the specific impact may be determined by the characteristics of the tumor and the patient's immune status.

In chronic hepatitis B, prolonged inflammation leads to liver fibrosis and progression to cirrhosis, ultimately leading to hepatocellular carcinoma. In patients with hepatitis and liver cirrhosis, the levels of IL-8 in serum and liver are significantly elevated. The increase in IL-8 may be related to the progression of liver cirrhosis, but current evidence suggests that the increase in IL-8 is not related to liver cirrhosis due to liver cancer [61, 62]. This evidence suggests that the fiber degradation protocol involved in IL-8 does not pose a safety risk for patients with liver cirrhosis. In our experiment, we used liver cirrhosis as the disease background and found that liver cirrhosis serum can activate the type I IFN signaling pathway of UC-MSCs, thereby improving treatment strategies. This method is mainly applied to the treatment of liver fibrosis and cirrhosis, rather than patients with liver cancer. Therefore, when conducting clinical transformation, it is necessary to strictly control the quality of enrolled patients and exclude patient at high risk of tumor development and those with cancer. In addition, the application of this therapy in patients with liver cirrhosis and liver cancer requires further experimental research, which is our team aim to carry out in future research.

## Conclusion

**Fig. 8:**
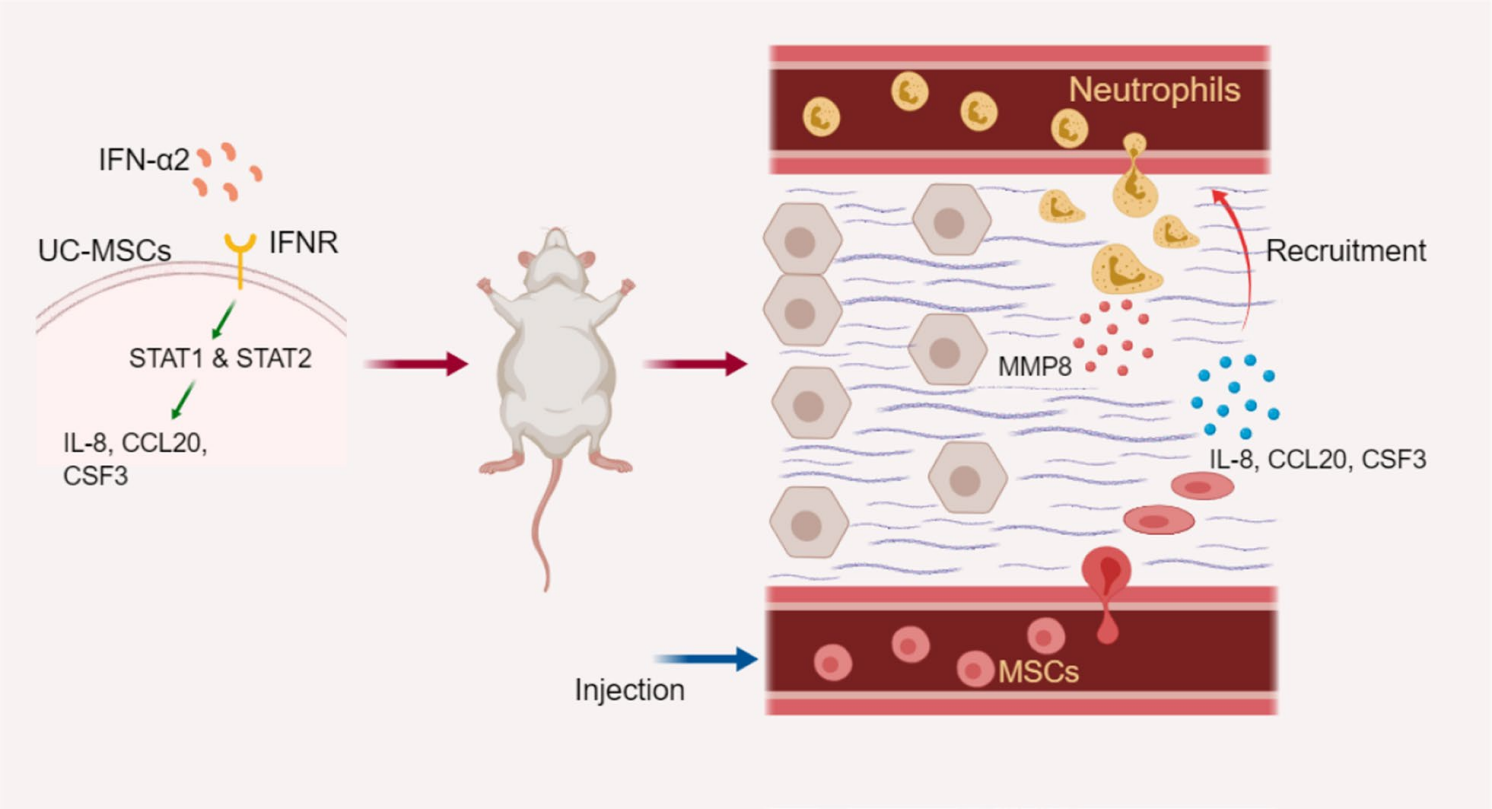
Potential mechanism of pretreated UC-MSCs with IFN-α2 to alleviate fibrosis. IFN-α2 induced UC-MSCs to secret more IL-8, CSF-3, and CCL20 through activating the p-STAT1 and p-STAT2 pathway. In vivo treatment, pretreated UC-MSCs recruited neutrophils to play a role, and the latter is the main source of MMP8

Our research demonstrates a new clinical use of UC-MSCs in the treatment of liver cirrhosis. UC-MSCs pre-treated with IFN-α2 showed a better therapeutic efficacy than untreated UC-MSCs. We observed the changes in the levels of expression of cytokines secreted by pre-MSCs and found that the STAT pathway was activated in these cells resulting in enhanced secretion of IL-8, CSF-3, and CCL20. Pre-MSCs recruited neutrophils, which are the main source of MMP8. This may represent the potential mechanism underlying the improved therapeutic effect of pre-MSCs (Fig. 8).

### Supplementary Information

**Additional file 1: Figure S1**. Characterization of UC-MSCs. **Figure S2**. Identification of UC-MSCs with IFN-α2 treatment biomarkers using flow cytometry. **Figure S3**. The effect of IFN-α2 on the UC-MSCs migration ability in vitro. **Table S1**. Primers used in the qRT-PCR analysis.

## Data Availability

The datasets of the current study are available from the corresponding author on reasonable request.

## Abbreviations

AST: Aminotransferase
ALT: Alanine transaminase
ALB: Albumin
CCl4: Carbon tetrachloride
CCL20: Chemokine (C–C motif) ligand 20
CSF-3: Colony-stimulating factor 3
DEGs: Differentially expressed genes
FBS: Fetal bovine serum
GO: Gene Ontology
HE: Hematoxylin and eosin
HRP: Horseradish peroxidase
IFN: Interferon
IFNAR1: IFN α/β receptor subunit 1
IL: Interleukin
KEGG: Kyoto Encyclopedia of Genes and Genomes LC, liver cirrhosis
MMP: Matrix metalloprotease
MPO: Myeloperoxidase
MSCs: Mesenchymal stem cells
PBS: Phosphate buffered saline
PCR: Polymerase chain reaction
PMN: Polymorphonuclear leukocyte
p-STAT1: Phosphorylated STAT
RNA-seq: RNA sequencing
shRNA: Short hairpin RNA
STAT: Signal transducer and activator of transcription
TBiL: Total bilirubin (TBiL)
TIMP1: Tissue inhibitors of metalloproteinase 1
TNF: Tumor necrosis factor
UC-MSCs: Umbilical cord-derived MSCs
