# Supplementary material for: Pretreatment of UC-MSCs with IFN-α2 improves treatment of liver fibrosis by recruiting neutrophils
Source: J Transl Med. 2023 Nov 18;21:832. doi: 10.1186/s12967-023-04732-0 (PMC10656886; doi:10.1186/s12967-023-04732-0)
Supplement: Supplementary file 1 — Additional file 1: Figure S1. Characterization of UC-MSCs. Figure S2. Identification of UC-MSCs with IFN-α2 treatment biomarkers using flow cytometry. Figure S3. The effect of IFN-α2 on the UC-MSCs migration ability in vitro. Table S1. Primers used in the qRT-PCR analysis. [file 12967_2023_4732_MOESM1_ESM.docx]

**Additional file 1**


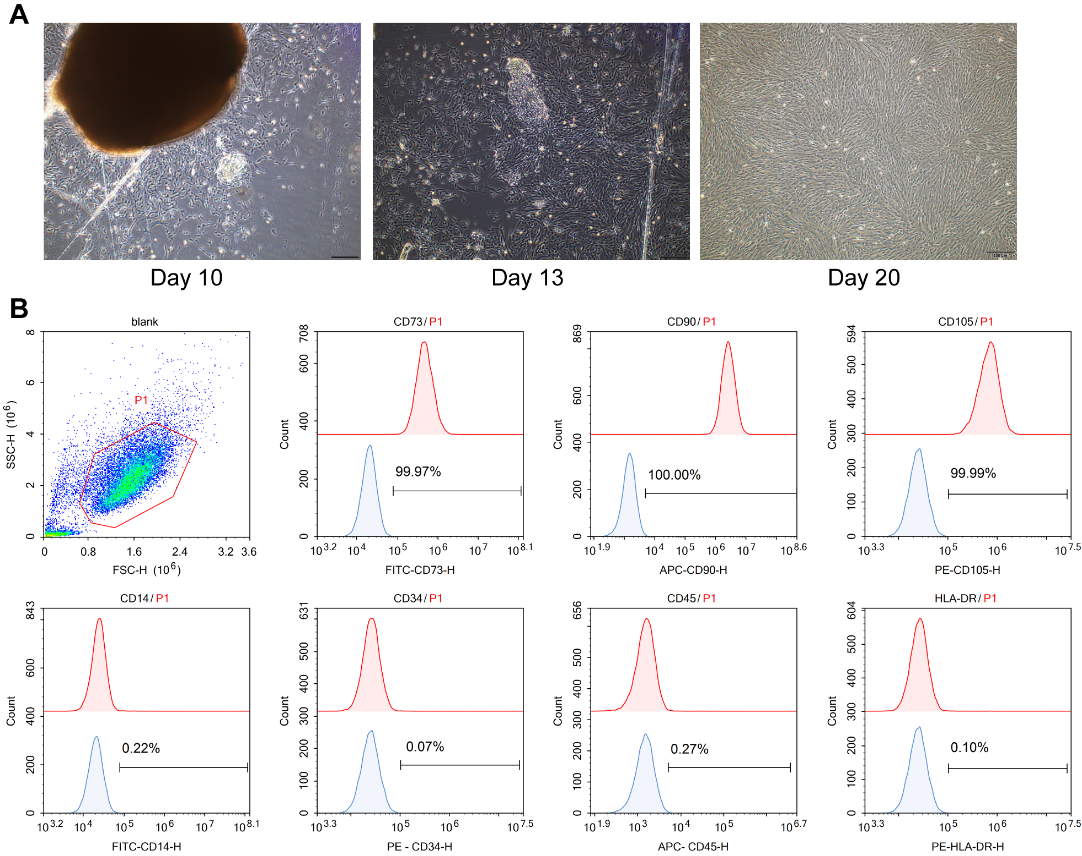


**Figure S1 Characterization of UC-MSCs.** A. Microscopy images of cells morphological of UC-MSCs. Scale bars: 100 µm. B. Representative flow cytometry plots showing UC-MSCs expressing the surface markers CD73, CD90, and CD105 but not CD14, CD34, CD45, and HLA-DR.


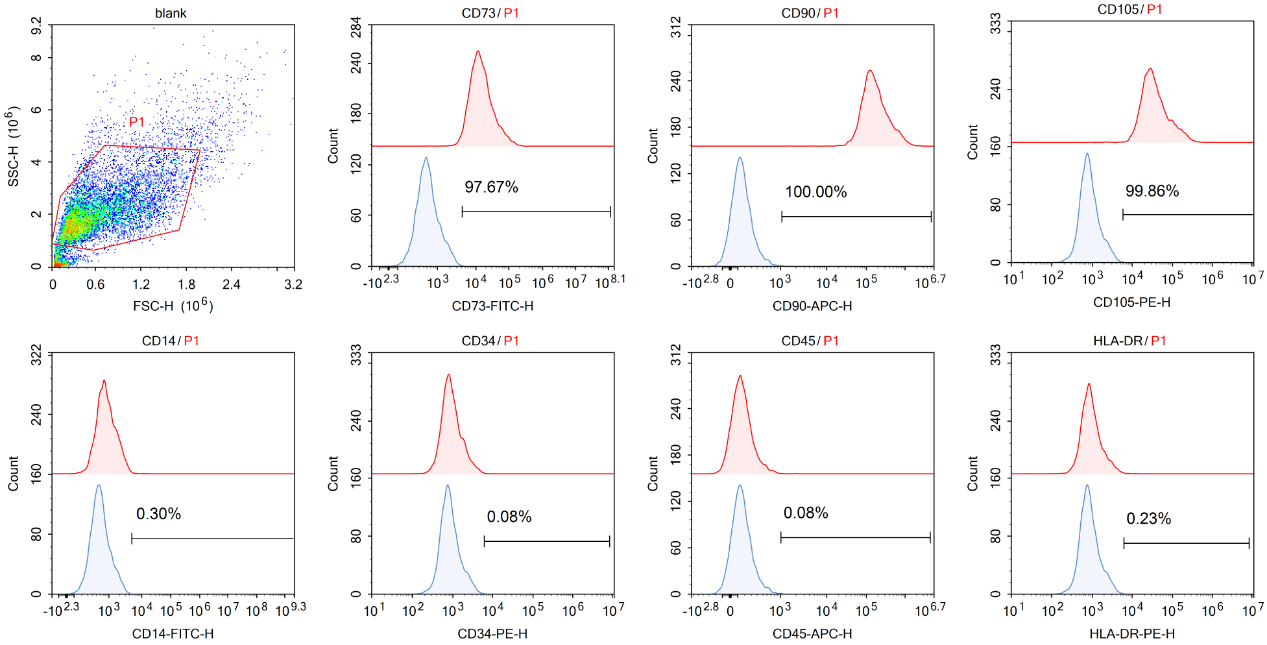


**Figure S2 Identification of UC-MSCs with IFN-α2 treatment biomarkers using flow cytometry.** Pre-MSCs expressing the surface markers CD73, CD90, and CD105 but not CD14, CD34, CD45, and HLA-DR.


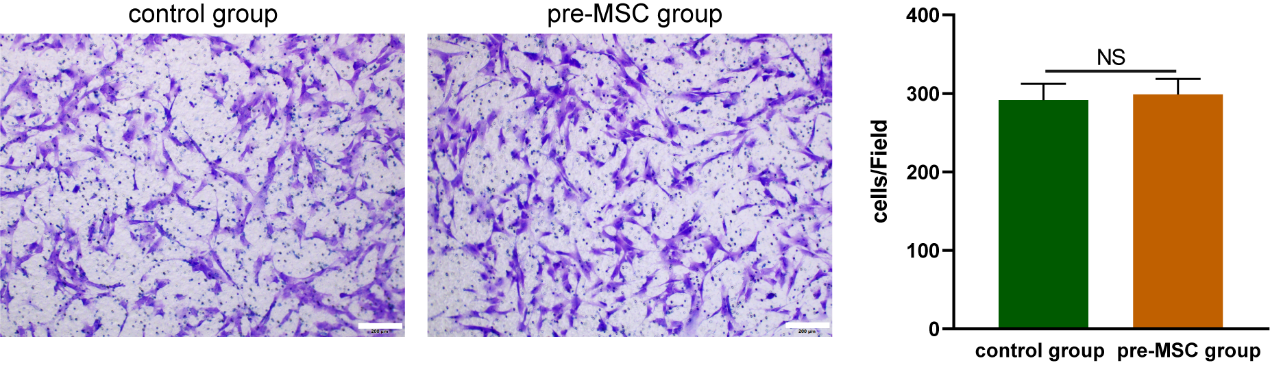


**Figure S3 The effect of IFN-α2 on the UC-MSCs migration ability in vitro.** Representative field of positive cells (Left) and analysis of the selected fields (Right). NS, no significance.

| **Table S1** Primers used in the qRT-PCR analysis | | |
| --- | --- | --- |
| Gene | Forward Primer | Reverse Primer |
| Human |  |  |
| GAPDH | TTGAGGTCAATGAAGGGGTC | GAAGGTGAAGGTCGGAGTCA |
| IL-1A | TGGTAGTAGCAACCAACGGGA | ACTTTGATTGAGGGCGTCATTC |
| IL-1B | TTCGACACATGGGATAACGAGG | TTTTTGCTGTGAGTCCCGGAG |
| IL-11 | CGAGCGGACCTACTGTCCTA | GCCCAGTCAAGTGTCAGGTG |
| TGF-β1 | CTAATGGTGGAAACCCACAACG | TATCGCCAGGAATTGTTGCTG |
| CXCL12 | ATTCTCAACACTCCAAACTGTGC | ACTTTAGCTTCGGGTCAATGC |
| CSF3 | GCTGCTTGAGCCAACTCCATA | GAACGCGGTACGACACCTC |
| CCL20 | TGCTGTACCAAGAGTTTGCTC | CGCACACAGACAACTTTTTCTTT |
| CXCL8 | ACTGAGAGTGATTGAGAGTGGAC | AACCCTCTGCACCCAGTTTTC |
| CSF1 | TGGCGAGCAGGAGTATCAC | AGGTCTCCATCTGACTGTCAAT |
| IFNAR1 | AACAGGAGCGATGAGTCTGTC | TGCGAAATGGTGTAAATGAGTCA |
| Rat |  |  |
| β-Actin | TGTGTTGTCCCTGTATGCCT | AATGTCACGCACGATTTCCC |
| MMP8 | CCCACCTGAGATTTGATGCT | GGATGCCGTCTCCAGAAGTA |
| MMP3 | AGACGGCCAAAATGAAGAGA | TTGTCCTTCGATGCAGTCAG |
| MMP9 | AGGGTCGGTTCTGACCTTTT | ATAAAAGGGCCGGTAAGGTG |
| MMP2 | TCCGAGTAAAGTATGGGAAC | CGTCACTCCGGCCAGTGTCT |
| MMP13 | TGACCTGGGATTTCCAAAAG | ACACGTGGTTCCCTGAGAAG |
| MMP14 | TGGGAACTTTGACACCGTGG | TTGGGTATCCGTCCATCACTTG |
| TIMP1 | TTCCCTGGCATAATCTGAGC | ATGGCTGAACAGGGAAACAC |
| TIMP2 | AAGATCACACGCTGCCCTAT | GTGCCCATTGATGCTCTTCT |

| **Table S2** Different expression of inflammatory factors | | | | | | | | |
| --- | --- | --- | --- | --- | --- | --- | --- | --- |
| Symbol | N-1 | N-2 | N-3 | C-1 | C-2 | C-3 | log2FC | regulated |
| CXCL1 | 75.25 | 53.82 | 75.82 | 8.08 | 7.44 | 5.11 | -3.13 | down |
| CXCL2 | 5.76 | 4.23 | 7.15 | 1.29 | 1.34 | 0.86 | -1.91 | down |
| CXCL3 | 7.31 | 3.71 | 7.11 | 1.18 | 1.00 | 1.34 | -1.85 | down |
| CXCL5 | 10.58 | 12.94 | 14.90 | 2.52 | 2.81 | 2.40 | -2.24 | down |
| CXCL6 | 16.31 | 11.19 | 15.73 | 3.10 | 4.03 | 3.08 | -1.98 | down |
| CXCL8 | 38.64 | 32.99 | 51.85 | 16.80 | 12.74 | 11.87 | -1.53 | down |
| IL1R1 | 24.18 | 25.15 | 26.96 | 5.95 | 7.21 | 5.47 | -2.03 | down |
| IL6ST | 75.84 | 77.86 | 77.04 | 49.83 | 51.00 | 50.04 | -0.71 | down |
| IL7R | 10.21 | 7.77 | 8.41 | 4.94 | 5.48 | 5.00 | -0.81 | down |
| NFIL3 | 14.07 | 14.96 | 16.90 | 10.44 | 8.82 | 9.87 | -0.73 | down |
| CCR10 | 1.26 | 0.72 | 1.02 | 1.57 | 2.24 | 2.39 | 0.65 | up |
| CXCL12 | 2.49 | 1.80 | 2.30 | 14.18 | 11.20 | 12.30 | 2.17 | up |
| IL11 | 8.64 | 4.19 | 9.16 | 15.86 | 13.67 | 15.39 | 0.87 | up |
| IL17RD | 0.38 | 0.47 | 0.33 | 0.89 | 0.66 | 0.78 | 0.74 | up |
| IL1A | 3.85 | 5.06 | 6.41 | 8.52 | 9.06 | 9.55 | 0.65 | up |
| IL1B | 10.16 | 14.49 | 18.15 | 45.20 | 46.11 | 38.46 | 1.38 | up |
| TGFBI | 373.73 | 360.74 | 321.15 | 638.34 | 615.39 | 583.94 | 0.67 | up |
| Data display is FPKM value | | | | | | | | |
